# The Conserved Roles of miR-2c in the Ecdysone Signaling Pathway by Targeting EcR/RXR and Runt for Exoskeleton Formation in the Pearl Oyster *Pinctada fucata martensii*

**DOI:** 10.3390/ani15233488

**Published:** 2025-12-03

**Authors:** Zhe Zheng, Weilin Gao, Yalin Xu, Hongmei Yang, Meichen Lu, Minxin Liang, Chuangye Yang, Jiawei Zhang

**Affiliations:** 1 Fishery College, Guangdong Ocean University, Zhanjiang 524088, China; zhengzhe@gdou.edu.cn (Z.Z.); 2 Pearl Breeding and Processing Engineering Technology Research Centre of Guangdong Province, Zhanjiang 524088, China; 3 Guangdong Science and Innovation Center for Pearl Culture, Zhanjiang 524088, China; 4 Guangdong Provincial Key Laboratory of Aquatic Animal Disease Control and Healthy Culture, Zhanjiang 524088, China; 5 Guangdong Provincial Engineering Laboratory for Mariculture Organism Breeding, Zhanjiang 524088, China

**Keywords:** miR-2c, ecdysone signaling, biomineralization, *Pinctada fucata martensii*, exoskeleton, chitin

## Abstract

The exoskeleton in gastropods and mollusks provides structural support, protection, and influences developmental processes. Although exoskeleton formation in gastropods is known to be regulated by the ecdysone signaling pathway modulated by miR-2, this mechanism remains poorly characterized in mollusk species. In this study, miR-2c was identified in the pearl oyster *Pinctada fucata martensii*, a commercially important bivalve, and was characterized as a conserved miRNA among gastropods and mollusks. In *P. f. martensii*, miR-2c was highly expressed in the mantle edge and during critical developmental stages, including the trochophore and D-shaped larval stages. In vivo experiments showed that overexpression of miR-2c disrupted crystal growth in both the prismatic and nacreous shell layers, whereas its inhibition altered shell repair processes. miR-2c directly targeted the 3′ untranslated regions (3′UTRs) of the ecdysone receptor (*EcR*), retinoid X receptor (*RXR*), and *Runt*-related transcription factors, which are central components of the ecdysone signaling pathway. Furthermore, miR-2c negatively regulated several downstream genes, including chitin synthase (*CHS*), *Runt*, and biomineralization-related factors (*AP*-*1*, *BMP7*, *KRMP*, *TYR2*). Sequence alignment revealed conserved miR-2c binding sites in *EcR*, *RXR*, *CHS*, and *Runt* across multiple species, indicating an evolutionarily conserved regulatory mechanism. These findings suggest that miR-2c coordinates the conserved regulation of core transcription factors *EcR*/*RXR* and *Runt*, serving as a key indicator of a common gene expression regulatory system for exoskeleton formation.

## Simple Summary

Exoskeletons (shells) help mollusks grow and survive, but how their formation is controlled is still not well understood. We studied a small regulatory RNA, miR-2c, in the pearl oyster *Pinctada fucata martensii* and found that it acts as a key switch linking hormone signaling to shell building. miR-2c is most active at the mantle edge and during early larval stages. When we increased miR-2c in vivo, crystal growth in both the prismatic and nacreous layers became abnormal; when we blocked it, shell repair changed. Laboratory assays showed that miR-2c directly binds the messenger RNAs of the ecdysone receptor (*EcR*) and retinoid X receptor (*RXR*), as well as the transcription factor Runt, and it reduces the activity of downstream shell-related genes such as chitin synthase. We also found conserved miR-2c binding sites in these targets across species, suggesting an evolutionarily shared control system for exoskeleton formation. Together, our results indicate that miR-2c coordinates ecdysone signaling and biomineralization genes to regulate shell growth. These insights provide candidate biomarkers and targets for breeding or management strategies aimed at improving shell/pearl quality and resilience in aquaculture.

## 1. Introduction

As organisms evolved from ancestral protists to multicellular animals, body size increased dramatically, necessitating the co-evolution of systems for movement and support. Two principal types of rigid skeletons have emerged for support: exoskeletons and endoskeletons [1,2]. Both skeleton types have undergone extensive evolutionary diversification, resulting in novel structures and morphologies adapted to a wide range of environments, thereby contributing to biodiversity. Notably, biomineralizations appeared subsequent to the emergence of skeletons; thus, the skeletal system is generally regarded as an independently evolving feature. The molluscan shell, exemplified by species such as the pearl oyster *Pinctada fucata martensii*, serves as a principal model system in biomineralization research. Although calcium carbonate (CaCO_3_) constitutes its primary mineral phase, the shell’s structural integrity and complex microarchitecture are dictated by an underlying organic matrix. Within this matrix, the polysaccharide chitin functions as an essential structural scaffold. The strategic use of chitin is an evolutionarily conserved mechanism, underpinning the formation of diverse skeletal structures across invertebrates, from the exoskeletons of crustaceans to the skeletal axes of certain ahermatypic corals such as black corals [3,4]. Critically, in the *Pinctada fucata*, chitin has been localized to the prismatic layer of the shell, and the gene encoding the key biosynthetic enzyme, chitin synthase (*CHS*), has been cloned and characterized [5]. These findings firmly establish the synthesis and deposition of chitin as a fundamental process in the assembly of the shell’s hierarchical crystalline organization. In contrast, collagen-based endoskeletons produced by osteoblasts form the axial and appendicular skeletons in vertebrates [6]. In vertebrates, skeletal growth is coordinated with overall body growth [7]. For gastropods and insects, exoskeletons impose constraints on body growth, necessitating periodic molting during the life cycle [8,9]. Interestingly, the exoskeletons of shelled mollusks such as bivalves exhibit indeterminate growth. Despite different growth models, chitin remains a common component of exoskeletons.

Chitin possesses a twisted plywood structure, composed of stacked planar arrays of complex chitin–protein fibers. In *scleractinian corals*, chitin constitutes a fundamental component of the organic matrix that guides biomineralization [10]. In arthropods, members of the Ecdysozoa, chitin is a major exoskeletal component and accompanying periodic molting by controlling its synthesis and degradation are tightly regulated in concert with molting cycles. In bivalves, the pearl oyster *Pinctada fucata martensii* is a model organism for shell biomineralization. The nacreous and prismatic shell layers consist of shells showed that the major components of organic matrices are chitin and proteins, such as Asp-rich calcium-binding proteins, nacrein, shematrin, and tyrosinase (*TYR*). These matrix components self-assemble extracellularly to form a framework that promotes biomineralization [5,11]. Disrupting the expression of chitin-binding proteins or chitinase alters CaCO_3_ crystallization and induces aberrant shell growth [12,13]. These findings indicate that chitin, together with the enzymes responsible for its synthesis and degradation, plays a critical role in calcified exoskeleton formation in mollusks.

In insects and crustaceans, the hormone ecdysone, recognized by the ecdysone receptor (EcR)–ultraspiracle (USP) complex, activates downstream signaling pathways that regulate essential physiological processes, including molting and growth, through precise control of chitin metabolism [14]. Ecdysone, also known as 20-hydroxyecdysone (20E), is a member of the ecdysteroid family and is widely recognized for its precise regulation of ecdysis—periodic exoskeletal remodeling accompanied by morphological changes—in arthropods. Ecdysone can diffuse across the plasma membrane and bind to intracellular receptors located in the cytoplasm or nucleus. The functional ecdysteroid receptor operates as a heterodimer, comprising the Ecdysone Receptor (EcR) and the Ultraspiracle protein (USP), with the latter serving as a functional ortholog of the vertebrate retinoid X receptor (RXR) [15]. In bivalves, including ecdysone is present in the serum and regulates the expression of target genes—such as chitin synthase (*CHS*)—via the EcR/RXR complex. Beyond *CHS*, this signaling pathway also modulates key biomineralization-related genes, including *KRMP* and Tyrosinase (*TYR*), as well as transcription factors like *Runt* and *AP-1*, which are implicated in shell matrix deposition and cellular differentiation [16]. A growing body of evidence further supports the role of ecdysone signaling in molluscan shell formation. For instance, transcriptomic analyses in the Pacific oyster *Crassostrea gigas* have revealed the involvement of ecdysone-related pathways during larval development and settlement, stages critical for shell biomineralization [17]. Collectively, these findings establish the ecdysone signaling pathway as a fundamental and evolutionarily conserved regulator of skeletal formation, functioning across both molting and non-molting invertebrates.

MicroRNAs (miRNAs) are endogenous small RNAs (~21 nucleotides) that negatively regulate gene expression by transcript cleavage or inhibition of translation. The miR-2c family is specific to invertebrates; for example, miR-2 is the largest microRNA family in *Drosophila melanogaster*, comprising eight members [18]. In arthropods, miR-2 modulates molting and metamorphosis by targeting ecdysone-mediated pathways, influencing cuticular protein and chitin biosynthesis as well as the juvenile hormone signaling pathway [19,20,21]. Recent studies have identified miR-2c in a variety of mollusc species, where it contributes to larval metamorphosis and biomineralization by targeting shell matrix proteins, growth factors, and core transcription factors, ultimately affecting shell formation [22]. For example, lgi-miR-2d is potentially associated with shell melanin synthesis via mitf targeting in Manila clam (*Ruditapes philippinarum*) [23]. In *P. f. martensii*, miR-2c is the primary miRNA bound by LncMPEG1, which negatively regulates biomineralisation-related genes such as *Notch*, *engrailed*, and *MMP* [20,24], that work was confined to describing this specific interaction and did not address the role of miR-2c in regulating the ecdysone signaling pathway. Therefore, our study aims to fill this critical knowledge gap by systematically investigating the functional link between miR-2c and the ecdysone pathway in the context of shell biomineralization.

As outlined above, ecdysone modulates chitin synthesis, and chitin is essential for constructing the organic matrix underpinning shell biomineralization [5]. Members of the *miR-2* family are well-established regulators of molting and metamorphosis, primarily through their targeting of genes within ecdysone-mediated pathways [19,20,21]. These observations suggest the possibility of miR-2-mediated regulation of chitin-based exoskeleton formation in bivalves via hormonal signaling pathways, although direct evidence has been lacking. In this study, a single copy of miR-2c was identified in. Through in vivo overexpression and inhibition experiments, it was demonstrated that miR-2c significantly affects shell growth. Furthermore, this study provides the first evidence that miR-2c directly targets *EcR* and *RXR* complex genes in the ecdysone pathway, together with its downstream target *PmRunt*. These findings reveal a conserved miRNA–hormone signaling regulatory network and provide insight into the mechanisms underlying exoskeleton formation in molluscs.

## 2. Materials and Methods

### 2.1. Experimental Animals

One-and-a-half-year-old pearl oysters (*P. f. martensii*) were obtained from Liushawan Farm, located on the coast of Liushawan, Zhanjiang (109°57′ E, 20°25′ N). Experimental oysters measured 5–6 cm in shell length and were maintained in circulating seawater at 25–27 °C for two days before experimentation.

Only oysters displaying robust shell condition and normal vitality were selected. After cleaning shell surface attachments, individuals underwent experimental treatments as required by each protocol. For tissue-specific expression analysis of miR-2c, the adductor muscle (A), mantle center (MC), mantle pallial (MP), mantle edge (ME), hepatopancreas (HE), gill (Gi), and foot tissues (F) were excised, immediately frozen in liquid nitrogen, and stored at −80 °C.

Adult *P. f. martensii* from a selectively bred population were induced to spawn by flowing water following brief air exposure. Fertilised eggs were cultured in 100 L buckets and subsequently transferred to 1000 L tanks, following the larval rearing protocol of Deng et al. [25]. Approximately 50,000 fertilized eggs were stocked in each 50 L tank, targeting an initial density of 1 egg/mL. As hatching rates are below 100%, larval density was monitored daily and meticulously maintained at approximately 1 individual/mL by adjusting the water volume through partial water changes, ensuring consistent density throughout the experiment. The larvae were fed at a total microalgal concentration of 40,000 cells/mL per tank. From Day 2 to Day 5, daily feeding consisted of *Isochrysis galbana* sp., followed by a mixture of *I. galbana* sp. and *I. zhanjiangensis* sp. from Day 6 to Day 50. Every other day, 300 L of filtered seawater was replaced in each tank. Water temperature was maintained at 25 ± 1 °C, with salinity at 30‰. The plastic film substrates were introduced into the tanks at the onset of the eye-spot larval stage to facilitate settlement. At approximately Day 50, juveniles measuring 2–3 mm were removed from plastic film substrates and transferred to 45 × 45 cm oyster nets at a density of 400 individuals per net. Developmental samples included unfertilised eggs (E) and eleven stages collected at 30 min, 5 h, 6 h, 8 h, 16 h, 19 h, 4 d, 14 d, 28 d, 40 d, and 90 d post-fertilisation. These represented fertilised eggs (Fe), blastocysts (B), gastrula embryos (G), early trochophore larvae (ET), trochophore larvae (T), D-type larvae (D), pre-feeding D-type larvae (DF), early umbo larvae (EU), eyespot larvae (EL), attachment larvae (S), and juveniles (J), larvae at specific developmental stages were collected using sieves, and each biological replicate consisted of approximately 500 individuals with three biological replicates per stage and replicates were defined as larval populations from the same batch.

### 2.2. In Vivo Modulation of miR-2c Expression

miR-2c mimics, miR-2c inhibitor, and negative control (NC) mimics were purchased from Genepharma (Shanghai, China). Fifteen specimens of Pf martensii, 1.5 years old, were randomly selected to three groups and injected in the adductor muscle with either miR-2c mimics, miR-2c inhibitor, or NC mimics at a dose of 20 μg/100 μL. After 72 h, MP tissues were collected from five individuals per group, rapidly frozen in liquid nitrogen, and stored at −80 °C for quantitative real-time polymerase chain reaction (qRT-PCR) analysis of miRNA and target gene expression. Total RNA was extracted from MP tissues using TRIzol™ Reagent (Thermo Fisher, Waltham, MA, USA). Residual tissue and debris on the inner shell surface were removed with double-distilled water (ddH_2_O). Shell fragments (approximately 1 × 1 cm, including prismatic and nacreous layers) were excised longitudinally, washed again with ddH_2_O, and air-dried. The inner shell surfaces were coated using an ion sputtering instrument. The crystal morphology of the nacreous and prismatic layers was observed and photographed under a scanning electron microscope (SEM).

### 2.3. qRT-PCR Validation

Total RNA was used to synthesise first-strand complementary DNA (cDNA) using the TransScript^®^ Uni All-in-One First-Strand cDNA Synthesis SuperMix for qPCR (One-Step gDNA Removal) kit (Transgen, Beijing, China). Subsequently, 2 µg of cDNA was used as template for quantitative RT-PCR with PerfectStart^®^ Green qPCR SuperMix (Transgen, Beijing, China) on a LightCycler^®^ 96 SW Quantitative RT PCR system. The cycling conditions were: pre-denaturation at 95 °C for 5 min; 95 °C for 10 s, 60 °C for 15 s, 72 °C for 15 s, for 45 cycles; 95 °C for 10 s, 65 °C for 60 s, 95 °C continuous; and 37 °C for 30 s cooling. Relative gene expression was analysed by the 2^−ΔCt^ and 2^−ΔΔCt^ methods [26,27]. Glyceraldehyde-3-phosphate dehydrogenase (*GAPDH*) was used as an internal reference gene. Primer sequences used in this study is shown in Table S1.

### 2.4. Seashell Damage Treatment

Fifty-six *P. f. martensii* of uniform size were selected. A V-shaped notch was cut along the longitudinal axis of the shell without damaging the mantle tissue. Mantle samples were collected at 0 h, 6 h, 12 h, 1 d, 1.5 d, 2 d, 3 d, and 5 d post-injury, with eight individuals sampled at each time point. All tissues were frozen in liquid nitrogen and stored at −80 °C.

### 2.5. Dual Luciferase Reporter Assay

Wild-type (WT) and mutant 3′-untranslated region (3′UTR) sequences of *RXR*, *EcR*, and *PmRunt* containing the predicted miR-2c binding sites were synthesised by Sangon Biotech (Shanghai, China) and cloned into the pmirGLO reporter vector, with plasmid preparation performed by Wuhan Transduction Biological Co., Ltd. (Wuhan, China) Endotoxin-free plasmids were extracted using the Endo-Free Plasmid Mini Kit II (Omega Bio-tek, Norcross, GA, USA). Target validation was conducted in HEK293T cells using a dual luciferase reporter assay. Cell culture and transfection followed standard protocols. Each group was transfected in triplicate with 100 ng plasmid, 50 nM miRNA, and 0.5 μL Lipofectamine 2000 (Thermo Fisher, Waltham, MA, USA) per well. The medium was changed 6 h post-transfection, and cells were cultured for 48 h prior to assay. Cells were washed twice with 500 μL phosphate-buffered saline (Thermo Fisher, Waltham, MA, USA), lysed with 100 μL 1 × PLB, and 20 μL cell lysate was used for luciferase activity measurements. Firefly luciferase activity was measured with 20 μL LAR II reagent, followed by renilla luciferase activity detection with 20 μL Stop & Glo reagent, following the manufacturer’s instructions for the Dual-Luciferase Reporter Assay System.

### 2.6. Sequences Collection and Target Prediction

Mature miR-2 family sequences were obtained from the miRBase database https://www.mirbase.org/ (accessed on 24 May 2025). Sequences of EcR (MW861535.1, XM_062078770.1, XM_070088599.1, XM_021350703.3, XM_027356260.2, XM_045275249.1), RXR (MW861536.1, XM_045248541.1, NM_001291921.2, NM_001011634.2, NM_001095479.1, NM_131238.1), Runt (KR825245.1, XM_048914871.2, XM_065492614.1, XM_064758297.1, NM_078700.4, AY508964.1, NM_001024630.4), and CHS (AB290881.1, AY291475.1, XM_028306879.1, XM_038021719.2, NM_079485.4) from *P. f. martensii* and related species were retrieved from the NCBI database, and their 3′UTRs were predicted. Potential miR-2c target sites within these 3′UTRs were predicted using the miRanda algorithm, and sequence alignments were performed to evaluate target conservation. Based on the *P. f. martensii* genome, potential precursor sequences of miR-2c were identified by local BLAST+ (version 2.14.0+). The secondary structure of each miR-2c precursor was predicted using M-fold, and a candidate precursor was considered valid if it could form a stem-loop structure with fewer than three base-pair mismatches in the stem region. Sequences used for multiple alignment were downloaded from miRBase database and aligned using ClustalW (v2.1). Precursor sequences from representative species.

### 2.7. Statistical Analysis

All data plots were generated using GraphPad Prism 6 (GraphPad Software, La Jolla, CA, USA). Values are presented as mean ± standard error of the mean (SEM), Homoscedasticity was verified using Levene’s test, and normality was assessed using the Shapiro–Wilk test. Student’s *t*-test was used for statistical analysis of dual luciferase reporter assays and target gene expression. One-way analysis of variance (ANOVA) was applied to identify the expression pattern of miR-2c and to analyze shell damage experiment results. All statistical analyses were performed using SPSS version 18.0 (IBM, Armonk, NY, USA). Differences were considered statistically significant at *p* < 0.05 or *p* < 0.01.

## 3. Results

### 3.1. Identification of miR-2c Precursor and Mature Sequence

**Figure 1:**
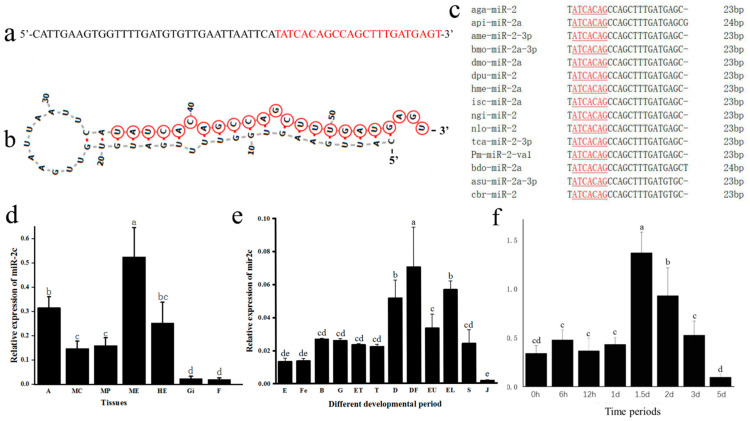
Identification and expression pattern analysis of miR-2c. (**a**) Precursor sequence of miR-2c, with the mature sequence indicated in red. (**b**) Predicted secondary structure of miR-2c; nucleotides in the red circle indicate the mature sequence. (**c**) Multiple sequence alignment of miR-2c mature sequences across species: aga, *A. gambiae*; api, *A. pisum*; bdo, *B. dorsalis*; dpu, *D. pulex*; tca, *T. castaneum*; nlo, *N. longicornis*; ngi, *N. giraultiisc*; isc, *Ixodes scapularis*; hme, *H. melpomene*; dmo, *D. mojavensis*; bmo, *B. mori*; ame, *A. mellifera*; Pm, *P. f. martensii*,; asu, *A. suum*; cbr, *C. briggsae*. Underlined letters indicate the seed sequence; numbers at right indicate mature sequence length. (**d**) Expression pattern of miR-2c in tissues: A, adductor muscle; MC, mantle center; MP, mantle pallial; ME, mantle edge; HE, hepatopancreas; Gi, gill. F, foot. (**e**) Expression during development: E, egg; Fe, fertilized ovum; B, blastocyst; G, gastrula; ET, early trochophore larva; T, trochophore larva; D, D-stage larva; DF, D-stage larvae prior to feeding; EU, early umbo larva; EL, eyed larva; S, spat; J, juvenile. (**f**) Expression of miR-2c after shell notching; different letters represent significant differences between the two groups (*p* < 0.05), and the same letters represent no significant differences between the two groups (*p* > 0.05).

Through sequence alignment, the precursor sequence of miR-2c are identified from the *P. f. martensii* genome (chromosome 2: g.82947865-g.82947811) (Figure 1a). The secondary structure, predicted using M-fold, displayed the typical stem-loop architecture characteristic of miRNAs, with the mature sequence located on the 3′ arm of the stem-loop (Figure 1b). Multiple sequence alignment demonstrated that miR-2c was completely identical to bases 1–20 and 22 of the selected sequences, with a highly conserved seed sequence (2–8 bp: “ATCACAG”) shared by all mature miR-2 family members. The mature miR-2c sequence exhibited 91.3% identity with homologous sequences from arthropod species (Figure 1c).

### 3.2. Differential Expression Analysis of miR-2c in Tissues and Developmental Stages of P. f. martensii

The expression analysis revealed distinct spatial and temporal patterns of miR-2c in *P. f. martensii*. In adult tissues, miR-2c showed significantly higher abundance in the mantle edge than in other tissues (*p* < 0.05). Moderate expression levels were detected in the adductor muscle, hepatopancreas, and mantle center, while the lowest expression occurred in the gill and foot (*p* < 0.05) (Figure 1d). During ontogenetic development across twelve stages from egg to larva, the expression profile displayed dynamic changes: miR-2c levels increased to a peak in D-stage larvae before feeding initiation, subsequently decreased significantly, and then showed a second expression peak at the eyed larval stage (*p* < 0.05). The juvenile period exhibited the lowest expression levels throughout development (*p* < 0.05) (Figure 1e).

### 3.3. Shell Damage Experiment

To investigate the role of miR-2c in shell formation, The shell damage experiment used a V-shaped notch, which damaged nacre and prismatic layers without damaging the mantle tissue and the mantle edges of the shell damage and control group were collected at eight temporal points. miR-2c expression was significantly upregulated at 1.5 days following shell notching (*p* < 0.05), then gradually returned to baseline by day 3 (Figure 1f). This expression pattern suggests that miR-2c plays a key role in shell reconstruction following damage.

### 3.4. Effects of miR-2c Overexpression and Inhibition on Shell Formation In Vivo

**Figure 2:**
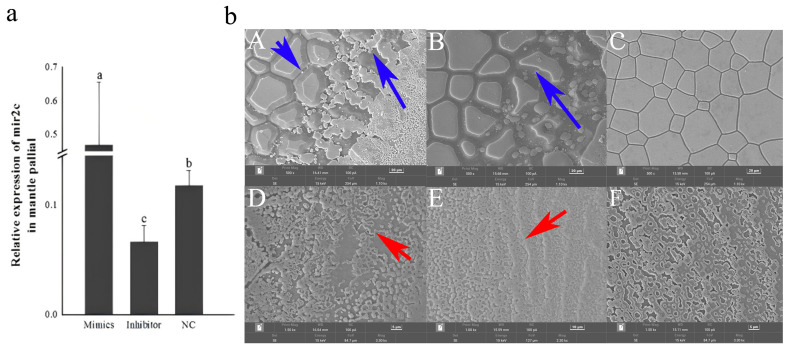
Effects of miR-2c overexpression and inhibitor on shell formation of *P. f. martensii*. (**a**) Expression of miR-2c after injection of mimics, inhibitor, or NC; different letters represent significant differences between the two groups (*p* < 0.05). (**b**) SEM images of prismatic layer (**A**–**C**) and nacreous layer (**D**–**F**) from oysters injected with mimics, inhibitor, and NC RNAs. Blue arrows indicate disordered prismatic layer growth; red arrows indicate disordered nacreous layer growth.

To assess the functional effect of miR-2c, mimics and inhibitors were injected to induce gain or loss of function in vivo. Compared with the NC, miR-2c expression in the was significantly increased by 1.27-fold following mimic injection, and significantly decreased by 1.71-fold in the inhibitor group (Figure 2a, *p* < 0.05). SEM analysis revealed that miR-2c overexpression promoted deposition in the prismatic layer, whereas inhibition of miR-2c increased the density of growth lines in the nacreous layer. Both overexpression and inhibition of miR-2c led to disordered crystal growth in both the nacreous and prismatic layers (Figure 2b).

### 3.5. Regulatory Role of miR-2c to EcR, RXR and Runt

**Figure 3:**
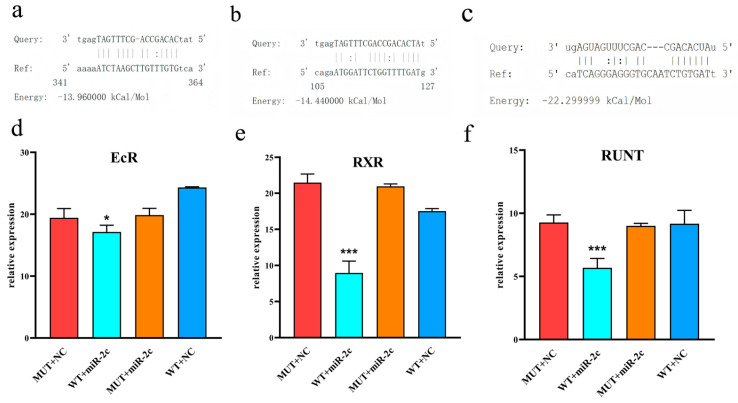
Effect of miR-2c on *EcR*, *RXR* and *Runt* genes detected by dual luciferase reporter system. (**a**) miRanda prediction of miR-2c targeting *EcR*. (**b**) miRanda prediction of miR-2c targeting *RXR*. (**c**) miRanda prediction of miR-2c targeting *Runt*. (**d**) Interaction between *EcR* and miR-2c; “*” indicates *p* < 0.05. (**e**) Interaction between *RXR* and miR-2c; “***” indicates *p* < 0.001. (**f**) Interaction between *Runt* and miR-2c; “***” indicates *p* < 0.001. WT: Wild-type-3′UTR pmir-GLO, NC: Negative Control mimic, miR-2c: miR-2c mimic, MUT: Mutant-type-3′UTR pmir-GLO, WT + miR-2c served as the experimental group, while MUT + NC, MUT + miR-2c, and WT + NC served as the control groups.

The dual luciferase reporter assay was used to assess the regulatory effect of miR-2c on the 3′UTRs of *EcR*, *RXR*, and *Runt*. Co-transfection of miR-2c mimics and pmirGLO-3′UTR constructs in HEK293T cells demonstrated direct targeting of these genes (Figure 3a–c). Compared with the MUT-3′UTR plus NC mimics group, luciferase activity in the EcR experimental group was significantly decreased by 1.13-fold (Figure 3d, *p* < 0.05), by 2.4-fold in the *RXR* group (Figure 3e, *p* < 0.001), and by 1.67-fold in the Runt group (Figure 3f, *p* < 0.001). miR-2c significantly inhibited the luciferase activity of WT 3′UTR constructs for *EcR*, *RXR*, and *Runt* (*p* < 0.05), whereas no significant effect was observed on the mutant (MUT) 3′UTR constructs (*p* > 0.05). NC mimics also had no significant effect on WT-3′UTR vector activity.

**Figure 4:**
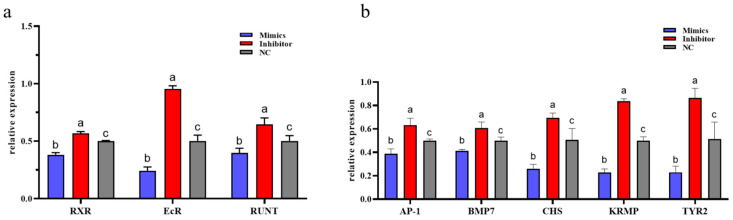
Expression of *EcR*, *RXR*, *Runt* and downstream genes after overexpression and inhibition of miR-2c. (**a**) Expression levels of *EcR*, *RXR*, and *Runt* following overexpression and inhibition of miR-2c. (**b**) Expression of genes downstream of the EcR/RXR pathway after miR-2c overexpression and inhibition; different letters represent significant differences between the two groups (*p* < 0.05).

To further validate this regulatory association in vivo, *EcR*, *RXR*, and *Runt* expression was assessed following overexpression and inhibition of miR-2c by exogenous injection of mimics and inhibitor. The relative expression levels of *RXR*, *EcR*, and *Runt* were significantly reduced in the mimics group compared with both NC and inhibitor groups (*p* < 0.05). In contrast, expression in the inhibitor group was significantly higher than in the NC and mimics groups (*p* < 0.05) (Figure 4a).

### 3.6. Effects of miR-2c to Downstream Genes of Ecdysone Pathway

Expression of downstream genes in the ecdysone signaling pathway—including *AP*-*1*, *BMP7*, *CHS*, *KRMP*, and *TYR2*—was also examined. Overexpression of miR-2c (mimics group) resulted in significant downregulation of these downstream genes (*p* < 0.05), with *BMP7* and *KRMP* showing highly significant reductions compared with NC (*p* < 0.01) (Figure 4b). Conversely, inhibition of miR-2c led to significant upregulation of all downstream genes compared with the NC group (*p* < 0.05).

### 3.7. Conservation Analysis of miR-2c Target Sites in EcR, RXR, CHS and Runt

**Figure 5:**
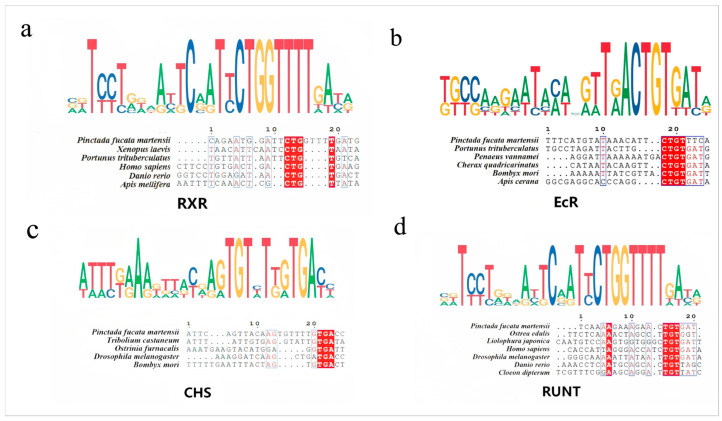
Sequence alignment of *RXR*, *EcR*, *CHS* and Runt between *P. f. martensii* and other species. (**a**) Alignment of miR-2c interaction sequence with *RXR*-3′UTR. (**b**) Alignment with *EcR*-3′UTR. (**c**) Alignment with *CHS*-3′UTR. (**d**) Alignment with *Runt*-3′UTR. Dark regions indicate 100% identical bases; lighter regions indicate strong similarity.

Homologues of *EcR* from *P. f. martensii*, *Portunus trituberculatus*, *Xenopus laevis*, *Apis mellifera*, *Homo sapiens*, and *Danio rerio* were collected, and target predictions for miR-2c were performed using miRanda. The interaction sites within the 3′UTRs of these genes were identified (Figures S1–S4), and sequence alignment revealed highly conserved regions. Similarly, conserved miR-2c target sites were observed at the 3′UTR of *RXR* genes from *P. f. martensii*, *P. trituberculatus*, *Penaeus vannamei*, *Cherax quadricarinatus*, and *Apis cerana*. Sequence comparisons of the *CHS* 3′UTR from *P. f. martensii*, *Ostrinia furnacalis*, *Bombyx mori*, *Tribolium castaneum*, and *Drosophila melanogaster* also demonstrated conservation of miR-2c binding sites. The *Runt* 3′UTR from *P. f. martensii*, *Danio rerio*, *D. melanogaster*, *H. sapiens*, *Liolophura japonica*, *Ostrea edulis*, and *Cloeon dipterum* showed conserved miR-2c interaction sequences (Figure 5).

## 4. Discussion

### 4.1. miR-2c Modulates Shell Formation in Larval and Adult Stages

The exoskeleton consists of skeletal elements with physical properties adapted to functional requirements and the eco-physiological constraints of the animal. Shelled molluscs form two types of shells during development: the prodissoconch is produced from the trochophore to metamorphic larval stages, whilst the adult shell forms post-metamorphosis. Although the prodissoconch and adult shell exhibit different morphology and ultrastructure, certain organic matrix components—such as chitin and matrix proteins—are present in both [28]. For example, proteomic analysis of blue mussel larval shells revealed that fibronectin type III, BPTI/Kunitz, and chitin-binding type 3 proteins, found commonly in adult shells, are found in larval shells as well [29]. In *P. f. martensii*, forty-nine nacre-specific genes are significantly upregulated at the trochophore stage [30]. Previous studies identified TYR and matrix metalloproteinase (*MMP*) as target genes of miR-2c [24]. TYRs, abundant in bivalve shell proteomes, are regarded as scaffold proteins that constitute the organic framework for biomineralizations.

In this study, we analyzed miR-2c expression at different developmental stages and observed a marked increase during the trochophore and metamorphic stages. This expression pattern suggests a regulatory role in both prodissoconch and adult shell formation, phases that are critically dependent on the precise secretion of shell matrix proteins and the establishment of shell microstructures [20,21]. The adult shell of *P. f. martensii* comprises two distinct calcium carbonate layers: an outer prismatic layer (comprising calcite) and an inner nacreous layer (comprising aragonite). This layered architecture is known to be synthesized by different regions of the mantle tissue, with the mantle edge primarily responsible for prismatic layer formation and the pallial/central mantle contributing to nacreous layer deposition [22,23]. Our functional experiments demonstrated that miR-2c activity directly influences this biomineralization process, overexpression of miR-2c in the mantle promoted prismatic layer deposition, whereas its inhibition increased the density of growth lines in the nacreous layer. The spatially restricted expression of miR-2c, which was significantly higher in the mantle edge than in the mantle center under physiological conditions, further supports the hypothesis that miR-2c primarily contributes to prismatic layer growth. This model is consistent with previous findings that the mantle edge is a hotspot for the expression of prismatic layer-specific matrix proteins [24]. The proposed role of miR-2c in prismatic layer construction was conclusively validated by our shell damage-recovery experiment. The temporal sequence of regeneration—where prismatic layer repair peaked at 1–1.5 d, preceding nacreous layer reconstruction, which showed a significant upregulation peaking at 24 h followed by a subsequent downregulation. This correlation strongly suggests that miR-2c is a key regulatory factor initiating prismatic layer biomineralization during both development and repair.

### 4.2. miR-2c Directly Targets Multiple Genes in the Ecdysone Signaling Pathway

Ecdysone, a class of ecdysteroids, regulates various developmental processes in arthropods from early embryogenesis through reproduction and adult stages. The functional ecdysteroid receptor is a heterodimer consisting of EcR and USP, an orthologue of the vertebrate RXR [31]. miRNAs play critical roles in modulating the ecdysone signaling pathway by targeting key signal transduction elements, including receptors. For example, miR-34-5p regulates ecdysteroid receptor (*EcR*) expression during tick salivary gland degeneration [32], and miR-14-3p regulates both EcR and E75 in the midgut of *Spodoptera litura* [33]. Although USP was not detected in bivalves, RXR was found. Previous work established that EcR and RXR can form a heterodimer to mediate ecdysone signaling in *P. f. martensii* [16]. ECR and RXR was considered to be the response elements of ecdysone to regulated shell growth, which was distinct to the vital hormone for the regulation of molting development and reproduction in arthropod. Our study found that *EcR* and *RXR* genes were the potential target genes. In this study, miR-2c negatively regulated *EcR* and *RXR* expression, as demonstrated by in vivo overexpression and inhibition experiments. Dual-luciferase reporter assays confirmed direct binding of miR-2c to the 3′UTRs of *EcR* and *RXR*, resulting in decreased reporter gene expression. These findings evidenced the negative regulation of miR-2c to *EcR* and *RXR* genes, therefore affecting the ecdysone signal activity via receptor heterodimers in pearl oyster.

In insects, such as the brown planthopper, miR-2a-3p modulates chitin biosynthesis in response to 20-hydroxyecdysone signaling by targeting phosphoacetylglucosamine mutase [34]. miR-71 and miR-263 jointly regulate CHS and chitinase to control locust molting. In our study, miR-2c overexpression and inhibition in vivo resulted in decreased and increased expression of *CHS*, respectively. A predicted miR-2c target site was identified at the 3′UTR of *CHS*, supporting the conclusion that *CHS* acts as a downstream gene of the ecdysteroid signalling pathway and is regulated by miR-2c.

**Figure 6:**
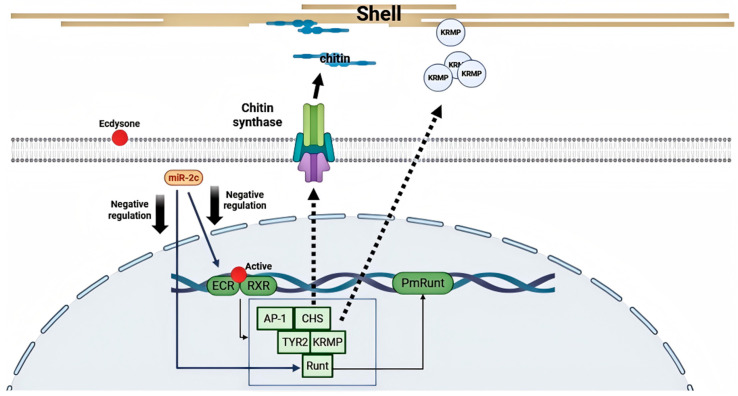
Regulation model of miR-2c on EcR/RXR pathway. The ecdysone-mediated signaling pathway, through the EcR/RXR heterodimer, activates PmRunt, AP-1, and other downstream biomineralization-related genes, including *CHS*, *KRMP*, and *TYR2*. miR-2c negatively regulates multiple genes within this signaling pathway, including *EcR*, *RXR*, and *PmRunt*, thereby affecting the expression of various effector genes responsible for exoskeleton formation.

Previous studies have indicated that biomineralization-related genes such as *AP*-*1*, *BMP7*, *Notch*, and *PmRunt* function downstream of the ecdysteroid signaling pathway. The Notch gene has been shown to be negatively regulated by miR-2c. In this study, we also identified a miR-2c target site in *PmRunt*. Both in vivo overexpression/inhibition and in vitro luciferase reporter assays confirmed that miR-2c negatively regulates *PmRunt* expression by directly binding its 3′UTR. *PmRunt* is a single-copy homologue of *RUNX2*, a key osteogenic factor in vertebrates. Growth factors such as IGF, FGF, and Notch mediate various signaling pathways that activate *RUNX2*, thereby affecting osteoblast proliferation and differentiation [35,36,37]. In pearl oyster, IGF and FATs are also closely associated with *PmRunt* activation [38]. Our results further support a regulatory effect of miR-2c on *PmRunt*, highlighting its pivotal role in biomineralization. Collectively, these findings indicate that miR-2c functions as a crucial regulator in exoskeleton formation by connecting multiple signaling pathways (Figure 6).

### 4.3. Evolutionary Footprint of miRNA Regulation Evidenced by Conserved Targeting Sites of miR-2c

miRNAs are a large class of small, non-coding RNAs present in plants and animals. Conservation of miRNAs across taxonomic lineages has been widely reported. Purifying selection has globally constrained the diversity of miRNA-containing regions and has strongly preserved the mature miRNA sequence [39]. The “seed” region of the miRNA (nucleotides 2–7) is the primary determinant of binding specificity and typically exhibits high conservation within the same miRNA family. In this study, we observed the conserved sequence of the miR-2 family. Beyond miRNA conservation, common target genes have also been identified across species. For example, the 3′UTRs of mouse and human Lin-28 genes contain extensive regions of sequence identity with sites complementary to the mammalian homologues of *C. elegans* lin-4 and let-7 microRNAs, indicating that microRNA regulation is a conserved feature of the Lin-28 gene in diverse animals [40]. Naidoo et al. identified strong conservation within the 3′UTR region complementary to the seed sequence of miR-71 [41]. However, limited conservation is observed in the regions flanking the seed complementarity sequence within the 3′UTR [42]. In this study, we collected the homologous genes of *RXR*, *EcR*, *CHS*, and *Runt* and performed target prediction for miR-2c binding sites. Conserved nucleotide sites were predominantly located at the seed region binding sites. Therefore, we propose that the regulatory capacity of the miR-2 family in exoskeleton formation in molluscs and arthropods, mediated by the ecdysteroid signaling pathway, may be conserved as an evolutionary footprint of miRNA regulation. The extraordinary phylogenetic conservation of miR-2c target sites, spanning from mollusks to deuterostomes, unveils a previously unrecognized and ancient regulatory stratum within steroid hormone signaling pathways [43]. Although ecdysone itself is a protostome-specific hormone, the fundamental partnership between its receptor (EcR) and RXR, as well as the core regulatory logic of this pathway, share deep evolutionary homology with vertebrate steroid hormone systems, particularly those involving RXR heterodimerization [44]. Our findings therefore suggest that miR-2c may represent of bioactive molecule with the potential for exogenously modulating endoskeletal systems.

## 5. Conclusions

In conclusion, miR-2c contributes to shell formation during both larval and adult stages and significantly affects the growth of the prismatic and nacreous layers in the adult shell. Furthermore, its transient upregulation during the prismatic layer regeneration phase in the shell repair model confirms its specific role in biomineralization. The conserved roles of miR-2c in the ecdysone signaling pathway were validated by its negative regulation of multiple genes, including EcR, RXR, and PmRunt, ultimately influencing the expression of various effector genes responsible for exoskeleton formation. These findings not only demonstrate the function of miR-2c-mediated regulation of the ecdysone signaling pathway, but also suggest that miR-2c may serve as an indicator of skeletal system regulation across species.

## Data Availability

The original contributions presented in this study are included in the article/Supplementary Material. Further inquiries can be directed to the corresponding author.

## Supplementary Materials

The following supporting information can be downloaded at: https://www.mdpi.com/article/10.3390/ani15233488/s1. Figure S1: Target prediction of miR-2c in RXR-3′UTR; Figure S2: Target prediction of miR-2c in EcR-3′UTR; Figure S3: Target prediction of miR-2c in CHS-3′UTR; Figure S4: Target prediction of miR-2c in RUNT-3′UTR; Figure S5: Shells for SEM; Figure S6: SEM images from all biological replicates; Table S1. Primer sequences.
