# Supplementary material for: The Conserved Roles of miR-2c in the Ecdysone Signaling Pathway by Targeting EcR/RXR and Runt for Exoskeleton Formation in the Pearl Oyster Pinctada fucata martensii
Source: Animals (Basel). 2025 Dec 3;15(23):3488. doi: 10.3390/ani15233488 (PMC12691275; doi:10.3390/ani15233488)
Supplement: Supplementary file 1 [file animals-15-03488-s001.zip › animals-3929561-v2.1-Supplementary materials.pdf]

The Conserved Roles of miR-2c in the Ecdysone Signaling Pathway by Targeting EcR/RXR and Runt for Exoskeleton Formation in the Pearl Oyster *Pinctada fucata martensii*

Zhe Zheng [1,2,3,4], Weilin Gao [1], Yalin Xu [1], Hongmei Yang [1], Meichen Lu [1], Minxin Liang [1], Chuangye Yang [1,2,3,5] and Jiawei Zhang [1,*]

1 Fishery College, Guangdong Ocean University, Zhanjiang 524088, China; zhengzhe@gdou.edu.cn (Z.Z.); 17520430487@stu.gdou.edu.cn (W.G.); 13662272418@stu.gdou.edu.cn (Y.X.); m18218045816@163.com (H.Y.); 18666019191@163.com (M.L.); 2112201065@gdou.edu.cn (M.L.); yangcy@gdou.edu.cn (C.Y.)

2 Pearl Breeding and Processing Engineering Technology Research Centre of Guangdong Province, Zhanjiang 524088, China

3 Guangdong Science and Innovation Center for Pearl Culture, Zhanjiang 524088, China

4 Guangdong Provincial Key Laboratory of Aquatic Animal Disease Control and Healthy Culture, Zhanjiang 524088, China

5 Guangdong Provincial Engineering Laboratory for Mariculture Organism Breeding, Zhanjiang 524088, China

* Correspondence: jiaweizhanggdou@gdou.edu.cn

Supplementary Materials

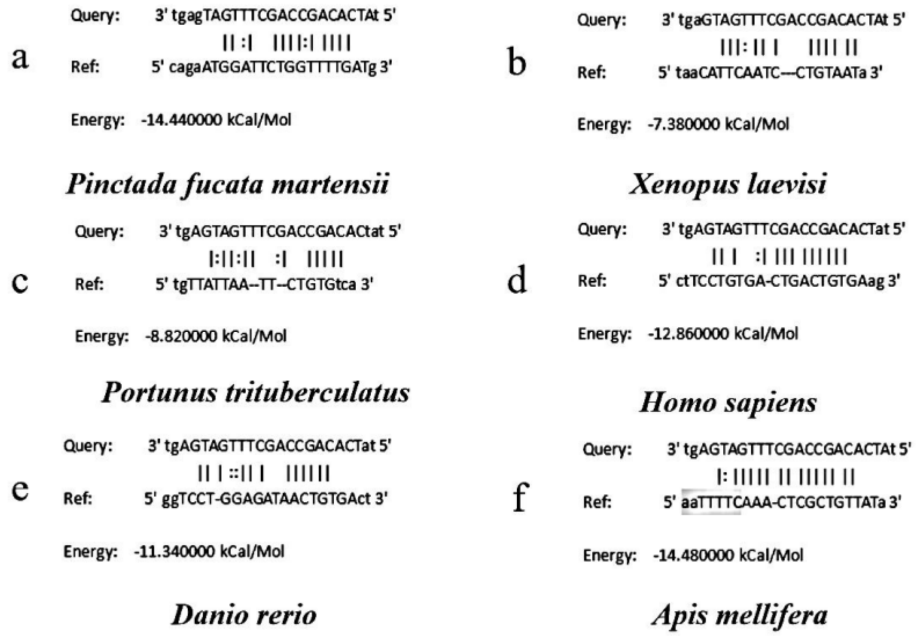

Figure S1 Target prediction of miR-2c in RXR-3'UTR. (a) Target prediction of miR-2c in *P.f. martensii* RXR-3'UTR. (b) Target prediction of miR-2c in *Xenopus laevis* RXR-3'UTR. (c) Target prediction of miR-2c in *Portunus trituberculatus* RXR-3'UTR. (d) Target prediction of miR-2c in *Homo sapiens* RXR-3'UTR. (e) Target prediction of miR-2c in *Danio rerio* RXR-3'UTR. (f) Target prediction of miR-2c in *Apis mellifera* RXR-3'UTR.

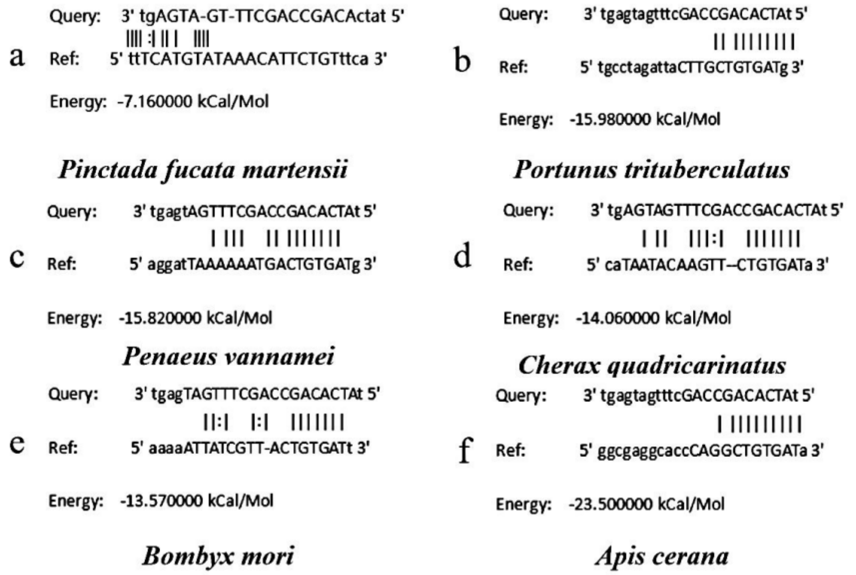

Figure S2 Target prediction of miR-2c in EcR-3'UTR. (a) Target prediction of miR-2c in *P.f.*

martensii EcR-3'UTR. (b) Target prediction of miR-2c in *Portunus trituberculatus* EcR-3'UTR. (c)Target prediction of miR-2c in *Penaeus vannamei* EcR-3'UTR. (d) Target prediction of miR-2c in *Cherax quadricarinatus* EcR-3'UTR. (e) Target prediction of miR-2c in *Bombyx mori* EcR-3'UTR. (f) Target prediction of miR-2c in *Apis cerana* EcR-3'UTR.

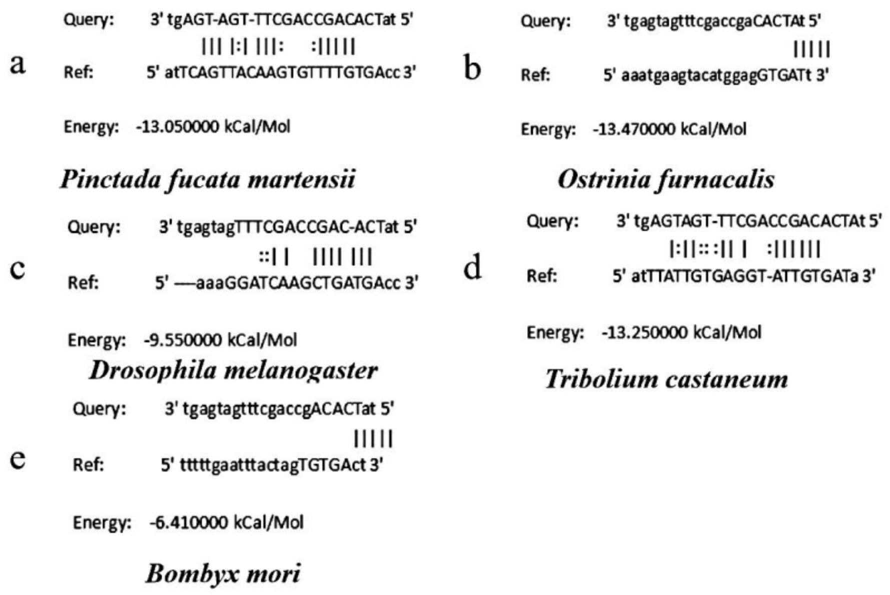

Figure S3 Target prediction of miR-2c in CHS-3'UTR. (a) Target prediction of miR-2c in *P.f. martensii* CHS-3'UTR. (b) Target prediction of miR-2c in *Ostrinia furnacalis* CHS-3'UTR. (c)Target prediction of miR-2c in *Drosophila melanogaster* CHS-3'UTR. (d) Target prediction of miR-2c in *Tribolium castaneum* CHS-3'UTR. (e) Target prediction of miR-2c in *Bombyx mori* CHS-3'UTR.

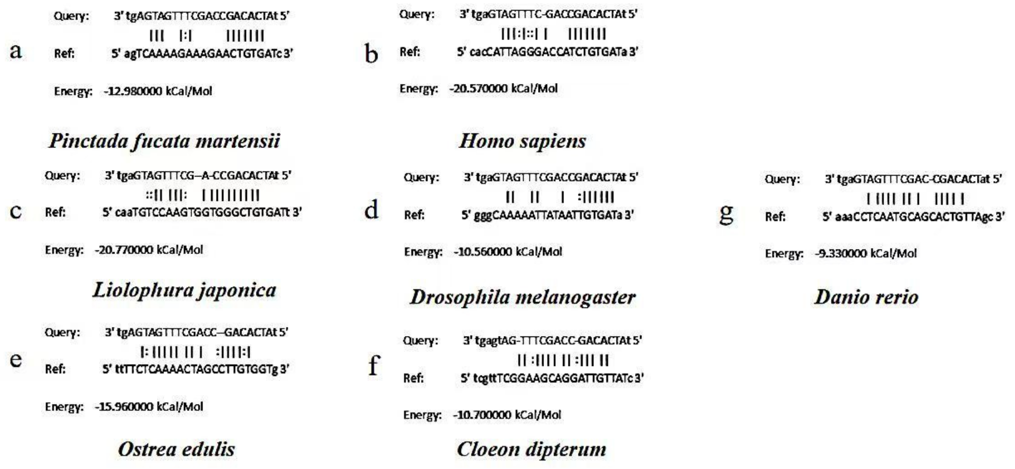

Figure S4 Target prediction of miR-2c in RUNT-3'UTR. (a) Target prediction of miR-2c in *P.f. martensii* RUNT-3'UTR. (b) Target prediction of miR-2c in *Homo sapiens* RUNT-3'UTR. (c)Target

prediction of miR-2c in *Liolophura japonica* RUNT-3'UTR. (d)Target prediction of miR-2c in *Drosophila melanogaster* RUNT-3'UTR. (e) Target prediction of miR-2c in *Ostrea edulis* RUNT-3'UTR. (f) Target prediction of miR-2c in *Cloeon dipterum* RUNT-3'UTR. (g) Target prediction of miR-2c in *Danio rerio* RUNT-3'UTR.

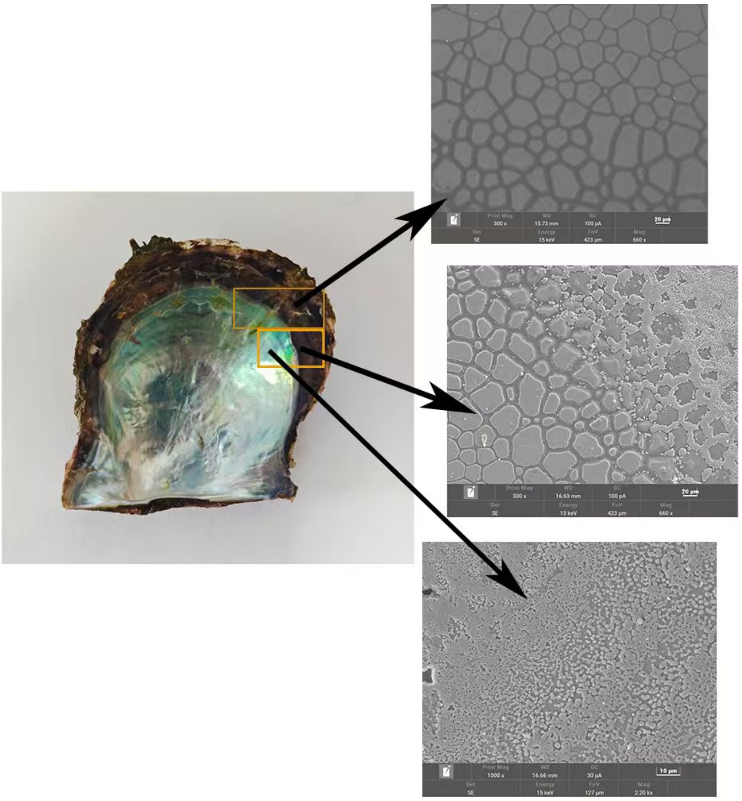

Figure S5 Shells for SEM.

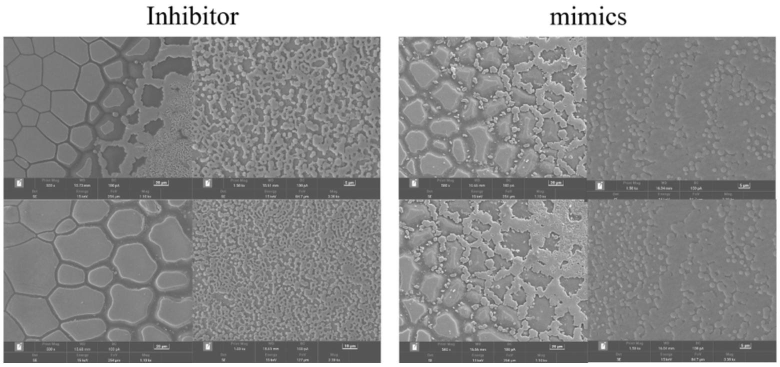

Fig S6 SEM images from all biological replicates.

Table S1. Primer sequences.

Primer name	Primer sequence (from 5'-3')	Application
PmEcR-S	GCTGGCTGTGGAACCTAAAAAC	qRT-PCR
PmEcR-A	CTATGGAGCGATGTGAAAATGC	qRT-PCR
PmRXR-S	CCAGGGTTTTCTCGTCTTTCAA	qRT-PCR
PmRXR-A	TGGCTGTCGGGTCTGTATCAT	qRT-PCR
PmRunt-S	TCCAGGACATCAAAGACCAGAAG	qRT-PCR
PmRunt-A	GGAACCAAACAATGGGCGA	qRT-PCR
AP-1-S	CGGTGGCAAAACAAAACAGC	qRT-PCR
AP-1-A	TGGGGCACGGGTAAAACTT	qRT-PCR
BMP2-S	TAACGAGGCTACTGGACACGAGA	qRT-PCR
BMP2-A	AGAATGTGAGGACGGTTTGCTT	qRT-PCR
TYR2-S	GGCATCGTGTTTATCTTGTGTTG	qRT-PCR
TYR2-A	CCATTGTGCTATCCCAGTAAGGTAT	qRT-PCR
KRMP-S	AAGAAATGTCACCCTTGGGATTGG	qRT-PCR
KRMP-A	AATCATCGCCACCATATCCATCG	qRT-PCR
CHS-S	ATCAGCCTGTTACCCCCG	qRT-PCR
Primer name	Primer sequence (from 5'-3')	Application
CHS-A	ACCTCTTCCTGTGCCTCATCT	qRT-PCR
GAPDH-S	GCAGATGGTGCCGAGTATGT	qRT-PCR
GAPDH-A	CGTTGATTATCTTGGCGAGTG	qRT-PCR
β-actin-S	CGGTACCACCATGTTCTCAG	qRT-PCR
β-actin-A	GACCGGATTCATCGTATTCC	qRT-PCR
